# School children’s mental health during the COVID-19 pandemic

**DOI:** 10.3389/fpsyg.2023.1290358

**Published:** 2024-01-05

**Authors:** Kristin Martinsen, Carina Lisøy, Tore Wentzel-Larsen, Simon-Peter Neumer, Lene-Mari Potulski Rasmussen, Frode Adolfsen, Anne Mari Sund, Jo Magne Ingul

**Affiliations:** ^1^Department of Psychology, Faculty of Social Sciences, University of Oslo, Oslo, Norway; ^2^Regional Center for Child and Adolescent Mental Health, Eastern and Southern Norway, Oslo, Norway; ^3^Regional Centre for Child and Youth Mental Health and Child Welfare - North, Faculty of Health Sciences, UiT, The Arctic University of Norway, Tromsø, Norway; ^4^Regional Centre for Child and Youth Mental Health and Child Welfare (RKBU), Department of Mental Health, Faculty of Medicine and Health Sciences, NTNU, Trondheim, Norway

**Keywords:** COVID-19, depression, anxiety, quality of life, school children

## Abstract

**Introduction:**

The COVID-19 pandemic significantly impacted the daily routines of children, with social distancing and quarantine leading to reduced social interactions and potential increased conflicts within families. These factors can increase the risk for anxiety and depression while reducing overall quality of life.

**Methods:**

Our study included 1843 school children aged 8 to 12 from 56 schools over a 2.5-year period before and during the pandemic. This multi-wave cross-sectional study utilized baseline data from an optimization trial of an indicated preventive intervention. The main outcomes were self-reported symptoms of anxiety and depression, and quality of life was the secondary outcome measure. Furthermore, responses to COVID-relevant questions were measured using a self-composed scale. Our objectives were to compare anxiety and depression symptom levels between cohorts of children who participated in the study before and during the pandemic, to examine if anxiety or depression predicted the COVID response, and whether anxiety and depression and subtypes of anxiety had an impact on quality of life during the pandemic. Linear regression and interaction models were used to examine relevant associations.

**Results:**

Levels of anxiety and depression were higher in all waves compared to pre-pandemic levels. Quality of life was lower during the pandemic than before the pandemic, particularly among children with generalized anxiety symptoms. Quality of life was negatively associated with loneliness.

**Discussion:**

Our study revealed that children reported higher anxious and depressive symptoms during the pandemic compared to pre-pandemic levels, as well as reduced quality of life. Lockdowns and restrictions may have contributed to this burden. Additionally, self-reported loneliness was a significant possible consequence of the restrictive measures imposed on children during the pandemic. Additional research is needed to investigate the long-term effects of the pandemic on children, particularly regarding the stability of elevated levels of anxiety and depression. Such studies could examine whether these conditions are indicative of a trajectory toward more severe internalizing disorders.

**Clinical trial registration:** NCT04263558.

## Introduction

Anxiety and depression are among the most prevalent mental health disorders among youths, are highly comorbid and co-occurring in a reciprocal pattern (Cummings et al., 2014; Long et al., 2018). According to a meta-analysis, the worldwide prevalence rates for anxiety and depression are 13.4 and 6.5%, respectively (Polanczyk et al., 2015), and prevalence rates have been rising (Lebrun-Harris et al., 2022). A recent meta-analysis of children’s mental health during the pandemic showed a pooled prevalence of 31% for both depressive and anxious symptoms (Deng et al., 2023). Another meta-analysis (Racine et al., 2021) assessing a total of 80,879 youth (mean age 13.0 years) globally showed a pooled prevalence for clinical elevated depressive symptoms of 25.2% and anxiety symptoms with 20.5% during the pandemic, which is twice as high as pre-pandemic estimates. Other reviews reporting prevalence rates for depressive and anxious symptoms during the pandemic have found similar results (Aarah-Bapuah et al., 2022; Oliveira et al., 2022). Elevated levels of depressive and anxious symptoms may cause decreased functional ability almost at the same extent.as having a full disorder (Loades et al., 2020). Avoidance of challenging situations and reduced social contact are typical strategies for children with symptoms of anxiety and depression. And indeed, during the COVID-19 pandemic, reduced social contact were the aim of preventive measures. Hence, the preventive strategies may have exacerbated already established maladaptive emotional responses in these youth and influenced vulnerable youth negatively.

The strict preventive measures during the COVID-19 pandemic resulted in substantial changes and disruptions in the everyday life of youth. Youth had to endure closed schools and stopped leisure-time activities, which led to social isolation, less peer contact and missed opportunities to practice and reach developmental milestones. Loades et al. (2020) reported that, in general, social isolation and loneliness in children and adolescents predicted an increase in symptoms of depression and possibly also anxiety. In fact, a study of the mental health consequences of COVID-19 in Europe found that youth were the most affected group (Di Fazio et al., 2022). Alteration in levels of depression and anxiety were most visible in the youth population.

Reduced contact with friends and teachers due to social distancing and quarantine, increased family conflicts and homeschooling are all risk factors for increased levels of anxiety and depression that are also associated with COVID-19 restrictions (Ravens-Sieberer et al., 2022) and are likely to affect youth quality of life. And indeed, Ravens-Sieberer et al. (2022) showed that reduced quality of life among youth was associated with COVID-19 along with increased rates of anxiety and symptoms of mental health problems in general. It is therefore reasonable to expect that the pandemic, where quarantine and lockdown led to social isolation, less interpersonal contact, and thereby increased loneliness, could result in increased levels of depressive symptoms and decreased quality of life. For anxiety, the picture is more unclear. As youth were less exposed to social performance situations and separations from caregivers during the pandemic, this could also result in an opposite outcome, temporary reduction in the expression of some anxiety problems (e.g., social anxiety and separation anxiety), accompanied by enhanced quality of life. However, it is also reasonable to expect an increase in anxious symptoms related to generalized anxiety characterized by uncontrollable worries, fear and increased arousal as the pandemic led to uncertainty across a wide range of domains and disruptions in daily life routines. Fear for health and worries related to parents’ job situation, all associated with COVID-19, could also lead to increased symptoms of generalized anxiety (Racine et al., 2021). Accordingly, during the time of the pandemic in fall of 2020 and winter of 2021, consultation volume in Norwegian primary care increased, before stabilizing at a higher level in 2021 (Evensen et al., 2022). The increase in consultations was highest for youth over 13 years of age with a 52.4% increase in symptoms of anxiety and depression in 2021 compared to the pre-pandemic years. In the specialist health care services these effects were manifested somewhat later (Evensen et al., 2022). Moreover, a nationwide survey in Norway for the years 2014–2021 (*n* = 227, 258; ages 13–18) showed that depressive symptoms were 2.13 percentage points higher than expected during the pandemic (von Soest et al., 2022).

In the current study, we examined self-reported level of anxiety and depression before and during the pandemic, and the responses to COVID specific questions and quality of life as reported by children aged 8–12 years participating in a preventive randomized factorial trial (for study information, see Neumer et al., 2021). The COVID specific questions were designed to gather information on how the child was dealing with the pandemic. Specifically, the questions addressed the child’s concerns regarding infection, any anxiety or worries escalating due to the pandemic, their feelings of loneliness, and their experiences with homeschooling. Since data were collected from school children at different grade levels in the five waves during 2.5 years of the pandemic, we were also able to examine the differential effects of the COVID pandemic as the pandemic progressed.

As children were exposed to several known risk factors for anxiety and depression (e.g., insecurity and loneliness) due to the pandemic, our objectives were to examine whether children who participated in the study during the pandemic reported higher symptom levels of anxiety and depression than children who participated before the pandemic started. Next (Long et al., 2018), whether the levels of anxiety or depression predicted COVID response (also see measures below) and whether this response changed as the COVID pandemic progressed. Furthermore (Cummings et al., 2014), whether self-reported quality of life depended on the general levels of anxiety and depression. We also investigated if specific anxieties (separation anxiety, social anxiety, or generalized anxiety) had a differential impact on quality of life during the pandemic. Finally (Polanczyk et al., 2015), we examined the relationship between quality of life, homeschooling, and loneliness. We hypothesized that the mitigation strategies imposed during the pandemic would lead to increased levels of anxiety and depression as well as reduced quality of life. Additionally, we anticipated that the responses to COVID-specific questions would predict the relationship between these variables. We also explored the impact of specific anxieties, homeschooling, and loneliness on quality of life.

## Methods

### Participants and procedure

The current study was cross-sectional and used baseline data from an optimization trial of an indicated preventive intervention (Clinicaltrials.gov NCT04263558 (11/02/2020); see also trial protocol, Neumer et al., 2021). Altogether, 56 public schools in urban and rural areas, from 30 municipalities across Norway took part. School children from grade four to six attended one of five waves during the intervention trial in the years 2020 through 2022 with a new grade level invited each semester. The participating children (*N* = 1843) had a mean age of 10.8 years (range 8 years and 1 month to 12 years and 9 months) (*SD* = 0.7), and 56% were girls (*n* = 1,039).

The recruitment process was carried out through several successive stages or waves. Children and parents at participating schools received oral and written information about the study, inviting children who experienced symptoms of anxiety or depression to participate. Children with valid parental consent completed electronic screening surveys at school and children who met the predetermined criteria for inclusion were subsequently enrolled in the study. Only data from T1 (Pre-intervention at each wave) have been used in the present study.

**Figure 1:**
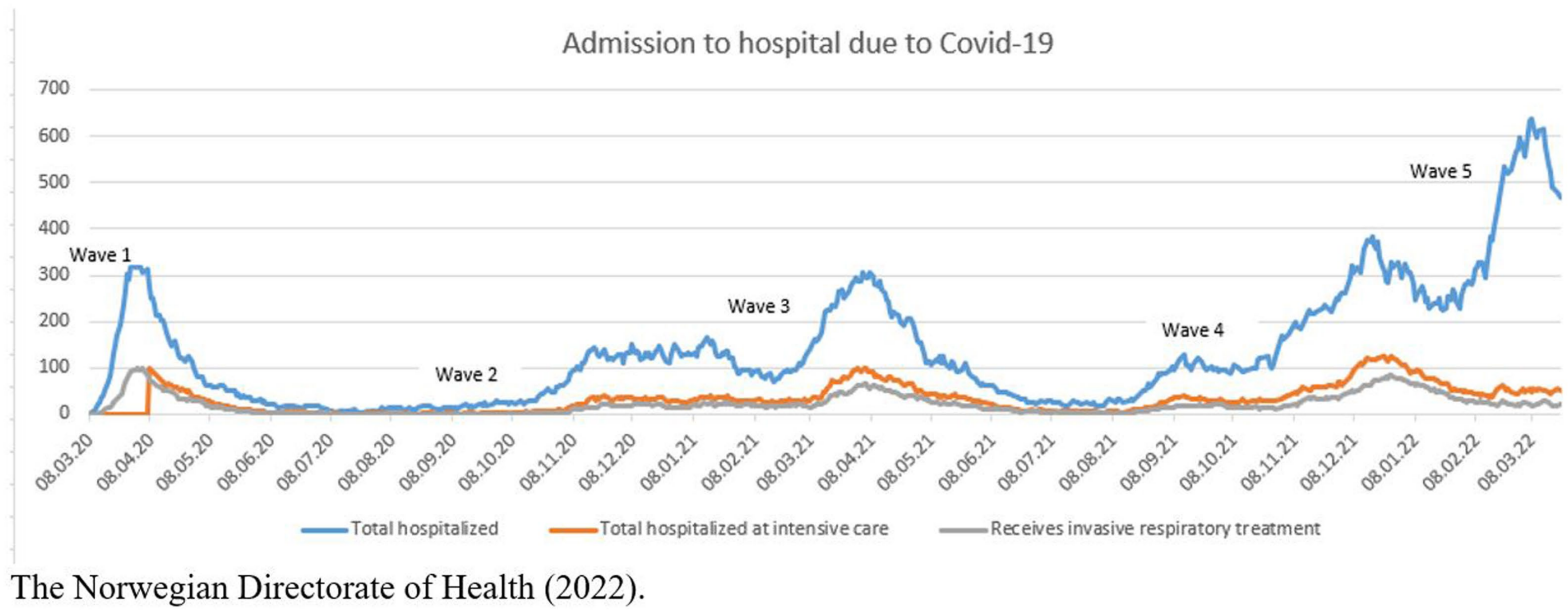
Number of people hospitalized with confirmed COVID in Norway March 2020–February 2022. The Norwegian Directorate of Health (2022).

The study started coincidentally just prior to the COVID-19-pandemic (early March 2020), and T1 data from the first data collection (Wave 1) were gathered before Norway was affected by the pandemic. In March 2020 the pandemic hit Norway, schools closed on a national level and infection control guidelines were initiated. As we were interested in children’s reactions to the pandemic and the restrictions imposed, we sought permission from the Regional Committees for Medical and Health Research Ethics (REK) – South East Norway to include questions in our survey concerning their experiences during the COVID-19 pandemic. Therefore, we classified data from the first wave of participants as “before the pandemic,” while data from the remaining four waves were classified as collected “during the pandemic.” The schools recruited children over five semesters, where data was collected in February 2020 (Wave 1), September 2020 (Wave 2), February 2021 (Wave 3), September 2021 (Wave 4) and February 2022 (Wave 5). The illustration below in Figure 1 depicts how the pandemic developed in Norway (The Norwegian Directorate of Health, 2022).

It is widely acknowledged that Norway was relatively mildly affected by the Corona virus when taking the population size into consideration. However, the measures imposed upon the population, not least upon the young ones, were stricter than in some neighboring countries, e.g., Sweden.

### Measures

The *Multidimensional Anxiety Scale for Children (MASC)* (March et al., 1997) is a self-report measure for children and youth 8–19 years, where 39 symptoms of anxiety are rated on a 4-point Likert-scale (0 = *never*, 1 = *rarely*, 2 = *sometimes* and 3 = *often*). A higher score indicates higher levels of anxiety, and the maximum total score is 117. MASC contains four subscales: Physical Symptoms, Social Anxiety, Separation Anxiety/Panic, and Harm Avoidance. The measure has demonstrated robust psychometric properties internationally (e.g., 18). Support for good internal consistency, structural validity, gender-based measurement invariance, convergent validity and diagnostic accuracy has also been demonstrated for the Norwegian version (Villabø et al., 2012; Martinsen et al., 2017). The Physical Symptoms subscale has been found to predict the presence of generalized anxiety (GAD) in children (Wei et al., 2014). Cronbach’s alpha coefficients for the total scale was.93, and alpha for the four subscales ranged from.66 (Harm Avoidance) to.89 (Social Anxiety and Physical Symptoms), consistent with previous Norwegian findings (Martinsen et al., 2017).

*The Mood and Feelings Questionnaire-short version (SMFQ)* (Angold et al., 1995) assesses depressive symptoms over the previous 2 weeks and is a 13-item self-report measure rated on a 3-point Likert-scale (0 = *not true*, 1 = *sometimes true*, and 2 = *true*) to assess core symptoms of depression in children and youth 6–18 years. Maximum total score is 26. Previous international studies have indicated that SMFQ is unidimensional (Messer et al., 1995), and has high internal consistency (Thabrew et al., 2018). Norwegian studies have also found high internal consistency (Lundervold et al., 2013; Martinsen et al., 2019), and support for structural and convergent validity (Lisøy et al., 2022). In the present study, Cronbach’s alpha for SMFQ was.90.

The *Kidscreen-27* (Ravens-Sieberer et al., 2007) contains 27 items derived from Kidscreen-52 (Ravens-Sieberer et al., 2005), measures generic health related quality of life for youth aged 8–18 on a 5-point scale (1 = “*not at all*” – 5 = “*very much*”). The scale has five dimensions, Physical Well-Being, Psychological Well-Being, Autonomy & Parents, Peers & Social Support, and School Environment. The total Kidscreen score is generated by summing up the responses. Higher scores on the measure indicate better quality of life (range raw scores, 27–135) (Ravens-Sieberer et al., 2005). Good psychometric properties of Kidscreen-27 have been demonstrated internationally (Befus et al., 2023) and in Scandinavia (Haraldstad and Richter, 2014). Support for the convergent and structural validity and reliability has also been documented specifically among Norwegian school children (Andersen et al., 2015). The Cronbach’s alpha for the scale was 0.93 in the present sample.

*COVID response.* To measure how the participants were influenced by the pandemic, a self-composed scale consisting of five items measured on a 5-point Likert-scale (1 = *not at all*, 5 = *very much*) was used. The items were (1) I have talked a lot with my parents (or the adults that I live with) about Corona (2) I have paid close attention to news about Corona, (3) I have worried about the Corona contagion, (4) Corona has made med more worried than I used to be, and (5) I felt lonely when schools were closed down. The five items were aggregated into a total score. Cronbach’s alpha for the five items was.72. I addition, respondents were asked about a single statement regarding homeschooling (“*Home-schooling has been a positive experience for me*”), which was also measured on a 5-point Likert-scale (1 = *not at all*, 5 = *very much*). COVID response variables were included for wave 2, 3 and 5. They were not included before the pandemic (wave 1) and COVID response variables were not collected for wave 4 due to technical error.

### Statistical analysis

To examine the level of symptoms of anxiety and depression during the COVID pandemic we used a linear regression model with the MASC and SMFQ, one at a time, as dependent variable, and time (wave 1 to wave 5) as a categorical covariate. A linear regression model was also used to examine whether levels of anxiety or depression were associated with COVID response. Here, COVID response was the dependent variable, while symptoms of anxiety and depression were independent variables.

To examine whether quality of life depended on general level of anxiety and depression and/or on specific anxieties (separation anxiety, social anxiety, or generalized anxiety), we first ran a regression analysis with quality of life as dependent variable and anxiety and depression as independent variables. Then, the specific anxieties were examined in a regression analysis with quality of life as dependent variable and separation anxiety, social anxiety, or generalized anxiety as independent variables.

Lastly, we examined quality of life and its relation to the children’s responses on issues relating to COVID, reactions to homeschooling and self-reported loneliness. In the first model, quality of life was the dependent variable and COVID response was independent variable. In a similar fashion, two separate models were run with quality of life as the dependent variable and experience with homeschooling and then loneliness as independent variable.

For all of the research questions above, we also ran interaction models to examine whether the relationships varied by participant wave. Missing values in the regression models were handled by complete case analysis. Effect size is reported using the partial Cohen’s f2 for continuous standardized regression coefficients with 0.02, 0.15, and 0.35 indicating small, medium and large effects, respectively, (Cinelli and Hazlett, 2020).

The statistical program IBM SPSS (version 22) was used for descriptive analyses. All regression models were conducted using R (The R Foundation for Statistical Computing, Vienna, Austria).

## Results

Altogether, 1843 children responded to the pre-intervention survey representing a response rate of 97, 98, 98, 97 and 91% in each wave of the children with consent (see Table 1). The participation rate, i.e., percentage of children with informed consent to participate in relation to the total number of children at the targeted age group at the schools, differed throughout the waves with more children screened in the first wave prior to the pandemic. In wave 1 through wave 5 the participation rate was 26, 12, 16, 13, and 9%, respectively. The low participation rate was expected as we recruited to an indicated study targeting children with elevated symptoms of anxiety and depression (see also Neumer et al., 2021). The reduction in children interested to participate as the pandemic progressed may be due to that the project and schools (who recruited the participants) may have communicated differently as the study progressed and the pandemic reached Norway. It was challenging for the schools to accommodate the present study within the mitigation strategies. It may also be the case that children and their parents were less interested in participating in such studies while there were pandemic restrictions. Mean scores on primary outcome measures of anxiety (MASC), depression (SMFQ), quality of life (Kidscreen-27 total raw score), and COVID response are shown in Table 1.

**Table 1:** Means and standard deviation (*SD*) across the five recruitment waves.

Measure	Wave 1 spring 2020 mean (SD)	Wave 2 fall 2020 mean (SD)	Wave 3 spring 2021 mean (SD)	Wave 4 fall 2021 mean (SD)	Wave 5 spring 2022 mean (SD)
MASC total	*n* = 693	*n* = 248	*n* = 344	*n* = 337	*n* = 221
	54.5 (20.2)	59.6 (18.6)	63.5 (18.6)	59.9 (18.8)	62.9 (19.0)
SMFQ total	*n* = 692	*n* = 247	*n* = 342	*n* = 336	*n* = 221
	8.0 (6.4)	8.7 (6.13)	9.8 (6.0)	8.8 (5.9)	9.0 (5.6)
Kidscreen-27 total	*n* = 693	*n* = 246	*n* = 343	*n* = 335	*n* = 215
	101.2 (17.3)	99.3 (17.0)	95.4 (16.1)	98.4 (16.1)	96.1 (15.2)
COVID response		*n* = 246	*n* = 343		*n* = 214
		12.4 (4.1)	12.8 (4.2)		11.9 (3.9)

SD = Standard Deviation; MASC = Multidimensional Anxiety Scale for Children; March et al., 1997, SMFQ = The Mood and Feelings Questionnaire-short version (Angold et al., 1995), COVID response: project developed measure in the ECHO-study, 2020.

### Symptom levels before, during and after the pandemic

There was a clear difference in anxiety symptoms between the waves, with a statistically significant difference observed (*p* < 0.001). Furthermore, there was also a significant difference for depressive symptoms between the waves (*p* < 0.001). While the means are reported in Table 1, the differences between the five waves are reported in Table 2. In all subsequent waves, participants reported higher levels of anxiety and depression compared to the pre-pandemic wave 1.

**Table 2:** Estimates of contrasts between waves for levels of anxiety (MASC) and depression (SMFQ).

MASC					SMFQ			
Contrasted waves	Coefficient	95% CI		*p*	Coefficient	95% CI		*p*
		LL	UL			LL	UL	
W 2–W 1	5.07	2.07	7.86	<0.001	0.76	−0.13	1.64	0.091
W 3–W 1	9.05	6.56	11.55	<0.001	1.89	1.09	2.68	<0.001
W 4–W 1	5.37	2.86	7.88	<0.001	0.82	0.03	1.62	0.043
W 5–W 1	8.42	5.48	11.35	<0.001	1.00	0.07	1.93	0.035
W 3–W 2	3.99	0.84	7.14	0.013	1.13	0.13	2.13	0.027
W 4–W 2	0.31	−2.86	3.47	0.850	0.07	−0.94	1.07	0.894
W 5–W 2	3.35	−0.16	6.86	0.062	0.25	−0.87	1.36	−0.666
W 4–W 3	−3.68	−6.58	−0.78	0.013	−1.06	−1.98	−0.14	0.024
W 5–W 3	−0.64	−3.91	2.63	0.702	−0.89	−1,92	0.15	0.095
W 5–W 4	3.04	−0.24	6.33	0.070	0.18	−0.87	1.22	0.739

MASC, Multidimensional Anxiety Scale for Children (March et al., 1997); SMFQ, Short Mood and Feelings Questionnaire (Angold et al., 1995). CI, Confidence Interval; LL, Lower Limit; UL, Upper Limit.

### Level of anxiety or depression and COVID response

Overall, there was a significant relationship between levels of anxiety and COVID response, (0.019, 95% CI [0.016, 0.023], *p* < 0.001). Effect size was calculated using Cohen’s partial f2. The effect was 0.14 which is almost in the medium range (small 0.02, medium 0.15). For symptom levels of depression however, the effect was not significant, (0.003, 95% CI [−0.008, 0.014], *p* = 0.631). COVID response was also examined separately per wave, see Table 3.

**Table 3:** Symptom levels and COVID response split by wave (*N* = 803).

Waves		Coefficient	95% CI		value of *p*
			LL	UL	
W 2	MASC	0.018	0.012	0.025	<0.001
	SMFQ	0.005	−0.015	0.026	0.607
W 3	MASC	0.019	0.013	0.024	<0.001
	SMFQ	0.015	−0.002	0.033	0.077
W 5	MASC	0.022	0.016	0.028	<0.001
	SMFQ	−0.027	−0.048	−0.005	0.014

Analysis on three waves with COVID response. W2: *N* = 246, W3: *N* = 343, W5: *N* = 221. CI = Confidence Interval, LL = Lower Limit, UL = Upper Limit. MASC = Multidimensional Anxiety Scale for Children (March et al., 1997). SMFQ = The Mood and Feelings Questionnaire-short version (Angold et al., 1995).

We found no evidence of an interaction between anxiety, COVID response and wave, *p* = 0.343. There were some indications that the relationship between depression and COVID response could depend on wave (*p* = 0.010), for details see Table 4. In wave 2 there was a very weak positive relation, in wave three, a weak positive relation, while in wave 5 there was a weak negative relation indicating that in this wave the COVID response decreased when depression increased.

**Table 4:** Symptoms of depression and COVID response split by wave (*N* = 803).

Waves		Coefficient	95% CI		*p*
			LL	UL	
W 2	SMFQ	0.005	−0.015	0.025	0.604
W 3	SMFQ	0.015	−0.001	0.032	0.073
W 5	SMFQ	−0.027	−0.048	−0.005	0.017

Analysis on three waves with COVID response, *N* = 803CI = Confidence Interval, LL = Lower Limit, UL = Upper Limit. SMFQ = The Mood and Feelings Questionnaire-short version (Angold et al., 1995).

### Quality of life during the pandemic

There was a significant overall difference in self-reported quality of life between the waves (*p* < 0.001). Results from examining whether the self-reporting of quality of life differed between waves are presented in Table 5. Results indicated that quality of life in waves 2–5, was below the level of quality of life reported in wave 1.

**Table 5:** Estimated contrasts between waves for levels of quality of life (*N* = 1,833).

Contrasted waves	Coefficient	95% CI		*p*
		LL	UL	
W 2–W 1	−1.98	−4.39	0.44	0.108
W 3–W 1	−5.80	−7.95	−3.65	<0.001
W 4–W 1	−2.83	−5.00	−0.66	0.010
W 5–W 1	−5.11	−7.65	−2.57	<0.001
W 3–W 2	−3.82	−6.54	−1.11	0.006
W 4–W 2	−0.85	−3.58	1.88	0.540
W 5–W 2	−3.13	−6.17	−0.10	0.043
W 4–W 3	2.97	0.47	5.47	0.020
W 5–W 3	0.69	−2.14	3.52	0.633
W 5–W 4	−2.28	−5.12	0.56	0.116

Quality of life measured by Kidscreen-27 total (Ravens-Sieberer et al., 2007). CI = Confidence Interval, LL = Lower Limit, UL = Upper Limit.

### Quality of life and general levels of anxiety and depression, and specific anxieties

Our data indicated significant relations between symptoms of anxiety and depression and quality of life, *p* < 0.001 indicating that quality of life decreased when anxiety and depression increased. For depression, the coefficient was −1.69, 95% CI (−1.81, −1.57). For anxiety we saw a smaller change, with a coefficient of −0.11, 95% CI (−0.15, −0.07). Cohens partial f2 indicated a small effect size of 0.02 for anxiety, and for depression the effect size was 0.42 which is considered large (>0.35).

Results concerning relations between the subscales of anxiety are reported in Table 6. The strongest support was for a relation between symptoms of generalized anxiety and quality of life, and there was some support for a relation between social anxiety and quality of life, while there was little support for such a relation to separation anxiety.

**Table 6:** MASC anxiety subscales and their relation to quality of life (*N* = 1,833).

	Coefficient	95% CI		*p*
		LL	UL	
MASC, Social anxiety	−0.17	−0.29	−0.06	0.00
MASC, Separation anxiety	−0.03	−0.15	0.10	0.685
MASC, Generalized anxiety (Physical)	−0.37	−0.49	−0.25	<0.001

MASC = Multidimensional Anxiety Scale for Children (March et al., 1997). Model adjusted for SMFQ. CI = Confidence Interval, LL = Lower Limit, UL = Upper Limit.

### Quality of life and COVID response, homeschooling, and loneliness

We then examined whether quality of life depended on COVID response as indicated by children’s mean response on the COVID questionnaire. We found a significant relation, where quality of life decreased when COVID response increased, coefficient = −3.90, 95% CI (−5.26, −2.54), *p* < 0.001).

Finally, we ran a regression analysis examining if experiences with homeschooling and self-reported loneliness were associated with quality of life, see Table 7. The results indicated that attitudes to homeschooling were associated with quality of life (*p* = 0.025). Examining the response alternatives “not at all” to the other response alternatives, it appeared that homeschooling *to some or to a large extent* has a relation to quality of life. For loneliness there was a clear relationship, (*p* < 0.001), where higher levels of loneliness during closed schools corresponded to lower quality of life.

**Table 7:** Attitudes toward homeschooling and loneliness, their relation to quality of life during the pandemic.

Contrasted response alternatives	Coefficient	95% CI		*p*
		LL	UL	
*Homeschooling (N* = 803)				0.025
«To a small extent» vs. “Not at all”	−0.08	−3.49	3.33	0.963
«To some extent» vs. “Not at all”	4.27	0.85	7.69	0.015
“To a large extent” vs. “Not at all”	3.540	−0.01	7.08	0.050
*«To a very large extent”* vs. “Not at all”	0.44	−3.28	4.16	0.817
*Loneliness (N* = 803)				<0.001
«To a small extent» vs. “Not at all”	−3.67	−6.46	−0.87	0.010
«To some extent» vs. “Not at all”	−4.99	−8.03	−1.94	0.001
“To a large extent” vs. “Not at all”	−9.67	−13.55	−5.79	<0.001
*«To a very large extent”* vs. “Not at all”	−14.95	−19.88	−10.02	< 0.001

Two of six self-developed questions regarding response to COVID. Analysis on three waves with COVID response, *N* = 803. CI = Confidence Interval, LL = Lower Limit, UL = Upper Limit.

## Discussion

The current study investigated how school children aged 8 to 12 years, who were screened for symptoms of anxiety and depression, responded during the COVID-19 pandemic in an indicated study. The objectives were to (1) examine whether children reported higher levels of anxiety and depression during the pandemic compared to children who participated before the pandemic, (2) whether anxiety or depression predicted COVID response, (3) if self-reported quality of life depended on the general levels of anxiety and depression and (4) how specific anxieties impacted quality of life during the pandemic. The study also explored the relationship between quality of life, homeschooling, and loneliness. It was anticipated that pandemic mitigation strategies would increase anxiety and depression, as well as decrease quality of life.

The findings revealed that children who responded during the pandemic had higher levels of anxiety and depression than those who responded before the pandemic which supported our hypothesis. Additionally, quality of life was also rated lower by the children during the pandemic than by the children who participated before the pandemic. Particularly for children with symptoms of generalized anxiety disorder who tend to worry a lot. Moreover, quality of life was also negatively associated with loneliness.

Children in all four participant waves during the pandemic reported higher levels of anxious symptoms than the children who participated just before the pandemic started. Mean levels of anxiety (MASC) ranged from 54.5 (SD = 20.2) to 63.5 (SD = 18.6) across the waves. This might indicate an increase in symptoms of anxiety during the pandemic.

Mean depressive symptoms across waves ranged from 8.0 (SD = 6.4) to 9.8 (SD = 6.0). This was higher than what has previously been found in a Norwegian sample of school children (mean age = 13.8, SD = 1.7), where the mean total score for SMFQ was 4.5 (SD = 4.7) (Larsson et al., 2016), hence the children in this indicated sample scored closer to a clinical level (Rhew et al., 2010).

The higher levels of depression and anxiety for those who responded during the pandemic compared to participants reporting in the wave before the pandemic, may be an effect of the pandemic. Other studies have indicated major post-pandemic increases in the prevalence of anxiety and depression among children and adolescents (Ravens-Sieberer et al., 2022). It has been suggested that this is a collective effect of the pandemic, whether it is due to the COVID infection itself or an impact of pandemic-related restrictions. However, as participants in the present study were new cohorts in each wave, we are not able to conclude that it was the pandemic that caused the increases.

Quality of life in the present sample was rated somewhat lower than in other studies with schoolchildren in the same age range (Berman et al., 2016; Budler et al., 2022). Lower quality of life ratings was expected, as this has previously been found in other similar samples Martinsen et al. (2016); Lehmann et al. (2023) and Ravens-Sieberer et al. (2023). Quality of life differed between the waves, and the children reported lower quality of life in all waves compared to the pre-pandemic participant wave. Again, study participants were different from wave to wave, so we cannot conclude that this was due to the COVID pandemic. However the high risk group in this sample and the fact that (Lehmann et al., 2023) recent studies have shown that the emotional well-being and quality of life for children declined during the initial year of the pandemic (Ravens-Sieberer et al., 2023) indicates that these children were at risk for severe consequences during the pandemic. Consequently, the extensive changes in the children’s daily lives, such as school closures and social distancing measures, may have come at a high cost for at risk children, who themselves were not at an elevated risk of severe COVID symptoms. As knowledge about the disease gradually emerged during outbreaks, it became apparent that socially disadvantaged children and those with mentally ill parents were particularly burdened, since the pandemic was especially a challenge for the whole family (Ravens-Sieberer et al., 2023). In this way, the pandemic had a strong and unique impact on family systems and may have taken the families’ buffer function away from children experiencing an extraordinary stressful time in life.

The current study showed that children with elevated symptoms of generalized anxiety, in particular, reported a decrease in their quality of life. These children reported feeling tense and experiencing somatic symptoms which are typical of generalized anxiety. Similar findings were also reported in the German study by Ravens-Sieberer et al. (2022), and a Norwegian study by Lehmann et al. (2023). Heightened generalized anxiety often sets off rumination. Living in a limited environment may restrict access to activities children often use to divert themselves and could thus result in reduced quality of life. Altogether, this adds to the heavy burden experienced by children during the pandemic, especially for those with elevated symptoms.

The study also showed that quality of life decreased with increased symptoms of depression and COVID response. Higher levels of loneliness during school lockdown were associated with lower quality of life. Previous research has established that loneliness in adults is linked to negative consequences for mental health and higher mortality rates (Hoffart et al., 2020). Furthermore, while older youth were able to maintain their relationships with peers through social media during lockdown, younger children may not have had easy access to these platforms. Studies have shown more loneliness among youth compared to older adults (Luhmann and Hawkley, 2016).

Attitude toward homeschooling was positively associated with quality of life, implying that children may have experienced fewer stressful situations such as separation from caregivers and coping with social challenging situations during the pandemic.

The study had several strengths. Children from both urban and rural schools reported on levels of anxiety, depression, and quality of life before and during the entire pandemic. Also well-established measures and sound statistical methods were used. Furthermore, the results are based on the reports from a large sample of at-risk young children, a group whose experiences is important to understand after an extended period of invasive infection control measures during the pandemic.

There were, however, also study limitations. Data were observational and collected from different participants at each wave, rather than following the same participants longitudinally over five waves. Hence, different participants contributed at different waves of the study, making comparisons across waves challenging. The participation rate was also higher in the pre-pandemic wave compared to in the following waves. Given that the target group was children exhibiting high levels of anxious and depressive symptoms, the lower participation rate was expected and intended. Nevertheless, employing more effective communication strategies could have allowed us to reach more children in the final waves. The sample was also high-risk and is thus not representative of children at large, limiting generalization and validity of the findings. Another limitation was the use of data from a single informant (children) based on self-report and not including multiple informants (children and caregivers). The use of self-report means we cannot rule out the possibility that response biases may have affected our results (Klein et al., 2018). The COVID response measure was self-developed based on face-validity, and psychometric properties (other than internal consistency) of the COVID response measure have not been documented.

To conclude, the at-risk sample of school children aged 8–12, reported elevated levels of anxiety and depression during the pandemic. The pandemic may have added to the symptom burden through lockdowns and social restrictions. Children reported higher symptom levels during the pandemic than what children did pre-pandemic. Similarly, children also reported reduced quality of life during the pandemic. Self-reported loneliness stood out as a possible serious consequence of the restrictive measures imposed on children. The current findings underscore the importance of health authorities exercising caution and careful consideration when implementing restrictions, particularly when it comes to children, in similar future situations. Furthermore, policy makers and health care workers should prioritize early interventions for children who are vulnerable to increased stressors due to their heightened levels of anxiety and depressive symptoms. By providing resources to these children, policymakers can help to prevent a decline in quality of life and mitigate long-term negative effects. Timely interventions can also reduce the burden on mental health services in the long run by preventing escalation of the symptoms and need for more intense treatment later in life.

Future studies should investigate long-term effects of the pandemic on these children to examine if such heightened levels of anxiety and depression are stable over time and if they are a trajectory to more serious internalizing disorders. Future research should also investigate interventions that can be accessed during periods of mitigation strategies, such as digital interventions.

## Data availability statement

Aggregate data may be available on request. Requests to access the datasets should be directed to k.d.martinsen@psykologi.uio.no.

## Ethics statement

The studies involving humans were approved by Regional Committees for Medical and Health Research Ethics (REK), (2019/1198) ref. 28762 ECHO. The studies were conducted in accordance with the local legislation and institutional requirements. Written informed consent for participation in this study was provided by the participants’ legal guardians/next of kin.

## Author contributions

KM: Conceptualization, Data curation, Formal analysis, Funding acquisition, Investigation, Methodology, Project administration, Writing – original draft, Writing – review & editing, Supervision. CL: Conceptualization, Data curation, Investigation, Methodology, Writing – original draft, Writing – review & editing, Visualization. TW-L: Formal analysis, Methodology, Writing – review & editing, Visualization. S-PN: Conceptualization, Data curation, Funding acquisition, Investigation, Methodology, Project administration, Writing – original draft, Writing – review & editing, Supervision. L-MR: Conceptualization, Data curation, Investigation, Methodology, Project administration, Writing – original draft, Writing – review & editing. FA: Conceptualization, Data curation, Funding acquisition, Investigation, Methodology, Project administration, Writing – review & editing. AS: Investigation, Writing – review & editing, Data curation, Methodology. JI: Conceptualization, Data curation, Funding acquisition, Investigation, Methodology, Project administration, Writing – original draft, Writing – review & editing, Supervision.
