# Corrigendum: School children's mental health during the COVID-19 pandemic

**DOI:** 10.3389/fpsyg.2024.1508141

**Published:** 2024-11-21

**Authors:** Kristin Martinsen, Carina Lisøy, Tore Wentzel-Larsen, Simon-Peter Neumer, Lene-Mari Potulski Rasmussen, Frode Adolfsen, Anne Mari Sund, Jo Magne Ingul

**Affiliations:** ^1^Department of Psychology, Faculty of Social Sciences, University of Oslo, Oslo, Norway; ^2^Regional Center for Child and Adolescent Mental Health, Eastern and Southern Norway, Oslo, Norway; ^3^Regional Centre for Child and Youth Mental Health and Child Welfare - North, Faculty of Health Sciences, UiT, The Arctic University of Norway, Tromsø, Norway; ^4^Regional Centre for Child and Youth Mental Health and Child Welfare (RKBU), Department of Mental Health, Faculty of Medicine and Health Sciences, NTNU, Trondheim, Norway

**Keywords:** COVID-19, depression, anxiety, quality of life, school children

In the published article, there was an error in Table 1 as published. The mistake identified was a miscode involving one of the items on the Kidscreen – 27 that was inadvertently reversed twice in the original dataset used for the paper mentioned above. This has led to an inconsistency in the published results for the rows involving Kidscreen-27 total. The corrected Table 1 and its caption appear below.

**Table 1:** Means and standard deviation (*SD*) across the five recruitment waves.

**Measure**	**Wave 1 spring 2020 mean (SD)**	**Wave 2 fall 2020 mean (SD)**	**Wave 3 spring 2021 mean (SD)**	**Wave 4 fall 2021 mean (SD)**	**Wave 5 spring 2022 mean (SD)**
MASC total	*n* = 693	*n* = 248	*n* = 344	*n* = 337	*n* = 221
	54.5 (20.2)	59.6 (18.6)	63.5 (18.6)	59.9 (18.8)	62.9 (19.0)
SMFQ total	*n* = 692	*n* = 247	*n* = 342	*n* = 336	*n* = 221
	8.0 (6.4)	8.7 (6.13)	9.8 (6.0)	8.8 (5.9)	9.0 (5.6)
Kidscreen-27 total	*n* = 693	*n* = 246	*n* = 343	*n* = 335	*n* = 215
	101.2 (17.3)	99.3 (17.0)	95.4 (16.1)	98.4 (16.1)	96.1 (15.2)
COVID response		*n* = 246	*n* = 343		*n* = 214
		12.4 (4.1)	12.8 (4.2)		11.9 (3.9)

SD, standard deviation; MASC, multidimensional anxiety scale for children (March et al., 1997); SMFQ, The mood and feelings questionnaire-short version (Angold et al., 1995); COVID response: project developed measure in the ECHO-study, 2020.

In the published article, there was an error in Table 5 as published. The mistake identified was a miscode involving one of the items on the Kidscreen – 27 that was inadvertently reversed twice in the original dataset used for the paper mentioned above. This has led to an inconsistency in the published estimated contrasts for levels of quality of life reported in Table 5. The corrected Table 5 and its caption appear below.

**Table 5:** Estimated contrasts between waves for levels of quality of life (*N* = 1,833).

**Contrasted waves**	**Coefficient**	**95 % CI**		***p***
		**LL**	**UL**	
W 2–W 1	−1.98	−4.39	0.44	0.108
W 3–W 1	−5.80	−7.95	−3.65	< 0.001
W 4–W 1	−2.83	−5.00	−0.66	0.010
W 5–W 1	−5.11	−7.65	−2.57	< 0.001
W 3–W 2	−3.82	– 6.54	−1.11	0.006
W 4–W 2	−0.85	−3.58	1.88	0.540
W 5–W 2	−3.13	−6.17	−0.10	0.043
W 4–W 3	2.97	0.47	5.47	0.020
W 5–W 3	0.69	−2.14	3.52	0.633
W 5–W 4	−2.28	−5.12	0.56	0.116

Quality of life measured by Kidscreen-27 total (Ravens-Sieberer et al., 2007). CI, confidence interval; LL, lower limit; UL, upper limit.

In the published article, there was an error in Table 6 as published. The mistake identified was a miscode involving one of the items on the Kidscreen – 27 that was inadvertently reversed twice in the original dataset used for the paper mentioned above. This has led to an inconsistency in the published MASC anxiety subscales reported in Table 6. The corrected Table 6 and its caption appear below.

**Table 6:** MASC anxiety subscales and their relation to quality of life (*N* = 1,833).

	**Coefficient**	**95 % CI**		***p***
		**LL**	**UL**	
MASC, social anxiety	−0.17	−0.29	−0.06	0.00
MASC, separation anxiety	−0.03	−0.15	0.10	0.685
MASC, generalized anxiety (physical)	−0.37	−0.49	−0.25	< 0.001

MASC, multidimensional anxiety scale for children (March et al., 1997). Model adjusted for SMFQ. CI, confidence interval; LL, lower limit; UL, upper limit.

In the published article, there was an error in Table 7 as published. The mistake identified was a miscode involving one of the items on the Kidscreen – 27 that was inadvertently reversed twice in the original dataset used for the paper mentioned above. This has led to an inconsistency in the published attitudes toward homeschooling and loneliness reported in Table 7. The corrected Table 7 and its caption appear below.

**Table 7:** Attitudes toward homeschooling and loneliness, their relation to quality of life during the pandemic.

**Contrasted response alternatives**	**Coefficient**	**95 % CI**		***p***
		**LL**	**UL**	
Homeschooling (*N* = 803)				0.025
≪To a small extent≫ vs. “not at all”	−0.08	−3.49	3.33	0.963
≪To some extent≫ vs. “not at all”	4.27	0.85	7.69	0.015
“To a large extent” vs. “not at all”	3.54	−0.01	7.08	0.050
*≪To a very large extent”* vs. “not at all”	0.44	−3.28	4.16	0.817
Loneliness (*N* = 803)				< 0.001
≪To a small extent≫ vs. “not at all”	−3.67	−6.46	−0.87	0.010
≪To some extent≫ vs. “not at all”	−4.99	−8.03	−1.94	0.001
“To a large extent” vs. “not at all”	−9.67	−13.55	−5.79	< 0.001
*≪To a very large extent”* vs. “not at all”	−14.95	−19.88	−10.02	< 0.001

Two of six self-developed questions regarding response to COVID. Analysis on three waves with COVID response, *N* = 803. CI, confidence interval; LL, lower limit; UL, upper limit.

In the published article, there was an error. The mistake identified was a miscode involving one of the items on the Kidscreen – 27 that was inadvertently reversed twice in the original dataset used for the paper mentioned above. This has led to an inconsistency in the published text, see below.

A correction has been made to **Measures**, third paragraph, last sentence. This sentence previously stated:

The Cronbach's alpha for the scale was 0.88 in the present sample. The corrected sentence appears below:

The Cronbach's alpha for the scale was 0.93 in the present sample.

A correction has been made to **Results**, first paragraph, last sentence. This sentence previously stated:

Mean scores on primary outcome measures of anxiety (MASC), depression (SMFQ), quality of life (Kidscreen-27), and COVID response are shown in Table 1.

The corrected sentence appears below:

Mean scores on primary outcome measures of anxiety (MASC), depression (SMFQ), quality of life (Kidscreen-27 total raw score), and COVID response are shown in Table 1.

A correction has been made to **Results** section, *Quality of life and general levels of anxiety and depression, and specific anxieties*, first paragraph. This sentence previously stated:

For depression, the coefficient was −1.58, 95% CI (−1.69, −1.47). For anxiety we saw a smaller change, with a coefficient of 0.09, 95% CI (−0.13, −0.06). Cohens partial f2 indicated a small effect size of 0.015 for anxiety, and for depression the effect size was 0.43 which is considered large (>0.35).

The corrected sentence appears below:

For depression, the coefficient was −1.69, 95% CI (−1.81, −1.57). For anxiety we saw a smaller change, with a coefficient of −0.11, 95% CI (−0.15, −0.07). Cohens partial f2 indicated a small effect size of 0.02 for anxiety, and for depression the effect size was 0.42 which is considered large (>0.35).

A correction has been made to **Results**, *Quality of life and COVID response, homeschooling and loneliness*, first paragraph. This sentence previously stated:

We found a significant relation, where quality of life decreased when COVID response increased, coefficient = −3.78, 95% CI (−5.05, −2.501), *p* < 0.001).

The corrected sentence appears below:

We found a significant relation, where quality of life decreased when COVID response increased, coefficient = −3.90, 95% CI (−5.26, −2.54), *p* < 0.001).

A correction has been made to **Results**, *Quality of life and COVID response, homeschooling and loneliness*, second paragraph. This sentence previously stated:

The results indicated that attitudes to homeschooling were associated with quality of life (*p* = 0.034).

The corrected sentence appears below:

The results indicated that attitudes to homeschooling were associated with quality of life (*p* = 0.025).

The authors apologize for these errors and state that this does not change the scientific conclusions of the article in any way. The original article has been updated.

